# VO_2_–Graphene Terahertz Multifunctional Metasurface with Switchable Broadband Waveplates and Absorber

**DOI:** 10.3390/nano16080490

**Published:** 2026-04-20

**Authors:** Hong Su, Tao Huang, Gaozhao Liu, Wentao Chen, Jiarong Zi, Chenglong Zhang, Shiping Feng, Min Zhang, Ling Li, Huawei Liang, Shixing Wang

**Affiliations:** State Key Laboratory of Radio Frequency Heterogeneous Integration, Key Laboratory of Optoelectronic Devices and Systems of Ministry of Education and Guangdong Province, Shenzhen Key Laboratory of Laser Engineering, College of Physics and Optoelectronic Engineering, Shenzhen University, Shenzhen 518060, China; hsu@szu.edu.cn (H.S.); 2300453066@email.szu.edu.cn (T.H.); 2400233029@mails.szu.edu.cn (G.L.); 2310452024@email.szu.edu.cn (W.C.); 2410232010@mails.szu.edu.cn (J.Z.); 2510232003@mails.szu.edu.cn (C.Z.); zhangmin@szu.edu.cn (M.Z.); liling@szu.edu.cn (L.L.); hwliang@szu.edu.cn (H.L.)

**Keywords:** graphene, metasurface, waveplate, absorber, terahertz

## Abstract

A terahertz multifunctional metasurface based on vanadium dioxide (VO_2_) and graphene that can switch between waveplate and absorber functionalities is proposed. As the temperature is below 300 K, by electrically controlling the Femi energy of the graphene it can realize half-wave plate (HWP) and quarter-wave plate (QWP) functionalities in the operating bandwidths of both 1.39–2.34 THz and 0.92–2.68 THz, respectively. While the temperature is above 340 K, the dipole resonance between VO_2_ and a gold reflector induces absorption. Furthermore, by applying the voltage to graphene, dual-parameter modulation of the amplitude of the transverse electric (TE) waves and the resonance frequency of the transverse magnetic (TM) waves is achieved, the absorption bandwidths of which are 3.65–3.78 THz and 1.41–3.12 THz, respectively. The operating frequencies for HWP, QWP, TE and TM waves can be tuned by changing the electrical field and working temperature. In addition, the incident angles are not sensitive to the performance of the metasurface, confirming its effectiveness even under large-angle incidence. The metasurface with simplicity in design, mature fabrication processes, and comprehensive functionality, has certain promising applications in terahertz optical switches, terahertz spectroscopy systems, modulators, and communication systems.

## 1. Introduction

In recent times, terahertz technology has generated substantial interest in applications such as detection [1], imaging [2], sensing [3,4,5], spectroscopy [6], and security devices [7]. Because of the lack of natural materials interacting directly with terahertz waves, multifunctional devices based on metamaterials have held promise and vast prospects for the development of terahertz technology, such as filters [8], modulators [9], waveplates [10], and so on. Among these investigations, terahertz waveplates and intensity modulators have emerged as particularly crucial components for applications in the terahertz regime. The generation of terahertz waves in lasers often results in waves with a single linearly polarized direction, whereas circularly polarized (CP) light is often more stable for imaging or transmission. Consequently, research on linear-to-circular (LTC) polarization conversion, cross-polarization conversion and amplitude conversion becomes essential in the context of terahertz applications in modulators and polarizers [11,12,13,14,15,16,17,18].

Integrating multiple functions into one device is in line with the further development trend. One approach is to combine the metasurface with both a two-dimensional material and a phase-change material for device design and thus the performance of the device can be tuned by applying external excitation. Metamaterials such as graphene [19,20,21,22,23,24], vanadium dioxide (VO_2_) [11,25,26,27,28], photosensitive silicon [29,30,31,32], and liquid crystals [33] have been utilized to create actively tunable devices through methods such as optical pumping, applied voltage and temperature modulation, thereby adjusting the material’s conductivity for terahertz applications [34,35]. By contrast, graphene has attracted increased attention from researchers due to its superior performance and its currently relatively mature production processes. Additionally, the optical property of graphene can be easily controlled by the gate voltage. This optical tunability enables the application of the graphene in terahertz modulators as an active layer. The high carrier mobility on the order of 10^6^ cm/Vs allows fast responses to electromagnetic fields [22]. Therefore, it serves as an excellent choice for integration into active metasurfaces. In 2023, Qingge Li et al. proposed a graphene-based absorptive metasurface in the gigahertz range with independent amplitude and frequency tuning. This metasurface allows dual-parameter modulation of both amplitude and frequency by adjusting the bias voltage applied to the graphene [23]. In 2021, W. Liu et al. proposed a dual-tunable metamaterial absorber based on a hybrid structure of VO_2_ and graphene, which can realize the transition between broadband and narrowband absorption [30]. Among the phase-change materials, VO_2_ is an exceptional material with a short response time, large modulation depth and various modulation approaches including electrical, thermal or optical excitation. Additionally, its electrical conductivity can be varied from 20 S/m to 2 × 10^5^ S/m by adjusting its temperature from 300 K to 340 K. In 2022, Lili Liu et al. proposed a tunable metasurface based on a VO_2_ layer and experimentally demonstrated its functionality in the terahertz band, achieving an absorption peak at 7.71 THz with potential applications in liquid and temperature sensing [28]. In 2023, Zhen Peng et al. reported a broadband absorption and polarization conversion switchable terahertz metamaterial device based on VO_2_ [11]. It is evident that the use of various actively tunable materials facilitates the integration of multiple functionalities within the same device, gradually becoming a prospective approach for the metasurface design of multifunctional terahertz devices, which is still significant in exploring how to fully exploit the structures of actively controlled materials and how to combine various active materials to achieve different functionalities.

In this paper, a metamaterial based on grating graphene and rectangular aperture VO_2_ structures for joint terahertz modulation is proposed. It can switch from a broadband absorber functionality to a multiple-waveplate functionality. Moreover, when the temperature exceeds 340 K, it can also simultaneously adjust both the amplitude of the TE polarization light and the resonant frequency of the TM polarization light by changing the bias voltage. The designed metasurface can not only act as a multiple-function terahertz device with absorber, half-wave plate and quarter-wave plate functionalities, but also realize dual-parameter modulation such as for amplitude and frequency.

## 2. Design and Simulation

**Figure 1:**
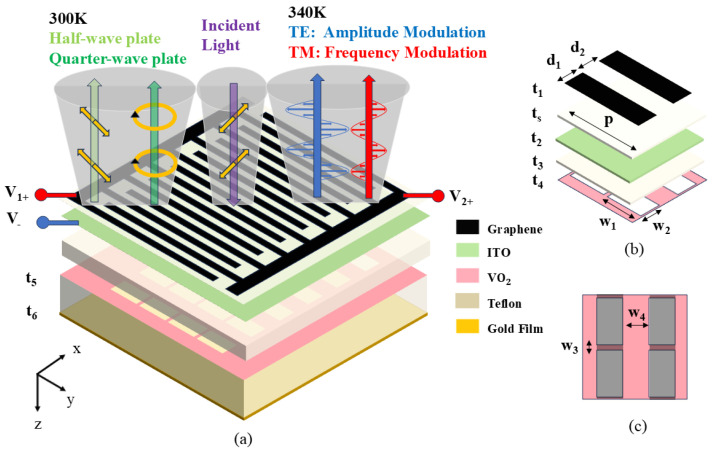
(**a**) Macroscopic schematic of a multifunctional terahertz modulator based on graphene and VO_2_. (**b**) Unit structure diagram. (**c**) Schematic diagram illustrating the relative positions of the graphene ribbons and VO_2_ in a unit cell.

The proposed multifunctional metasurface comprises a seven-layer structure arranged from top to bottom as follows: a four-layer graphene grating, silicon dioxide, indium tin oxide (ITO), polytetrafluoroethylene (PTFE), rectangular-aperture VO_2_, PTFE, and a gold reflective film. Their thicknesses are: t_1_ = 1.34 nm, t_s_ = 0.01 μm, t_2_ = 0.01 μm, t_3_ = 24.4 μm, t_4_ = 0.4 μm, t_5_ = 25 μm and t_6_ = 0.2 μm, respectively, as illustrated in Figure 1a. In Figure 1b, the distances between the adjacent graphene ribbons a-re set as d_1_ = d_2_ = 16 μm, with a single unit cell period p = 64 μm. The dimensions of the VO_2_ rectangular aperture are specified as follows: the length w_1_ = 28.8 μm, the width w_2_ = 16 μm, w_3_ = 3.2 μm and w_4_ = 16 μm. Figure 1c illustrates the relative positions of the graphene ribbons and rectangular apertures of VO_2_ in the same unit. In Figure 1a, the incident waves are set to propagate along the z-axis with the TE direction aligned with the x-axis or 45-degree angle along the positive x-axis directions. When the temperature is below 300 K, the modulator functions as a waveplate, enabling switching between a half-wave plate and a quarter-wave plate at the different bias voltages. However, it can be utilized as a dual-parameter modulator, modulating the amplitude of the TE waves and the absorption frequency of the TM waves as the temperature exceeds 340 K. Moreover, the designed graphene ribbons and rectangular apertures of VO_2_ have a mature production processes, making the fabrication relatively simple.

To facilitate the application of the different bias voltages to adjacent graphene ribbons, a staggered comb-like structure is designed for the graphene, allowing for flexible adjustment of the graphene layer’s periodicity. This structure is experimentally feasible via standard micro–nano fabrication processes: interdigital electrodes are patterned by photolithography, adjacent graphene ribbons are electrically isolated by dry etching, and the ITO layer acts as a common ground electrode for independent bias voltage application to each ribbon. In the terahertz range, the contribution of the intraband electrons plays a dominant role in the surface conductivity of graphene based on the Pauli exclusion principle, while the electronic interband transition is negligibly small when the Fermi energy *E_F_* ≫ *k_B_*T, here $k_{B}$, and T are the Boltzmann constant and the temperature of the graphene. Therefore the conductivity σ of the graphene can be simplified as the Drude model [36]:

(1) $$\sigma \left(\omega ,\tau ,E_{F}\right)=\frac{je^{2}E_{F}}{\pi \hbar^{2}\left(\omega +j\tau^{-1}\right)},$$

where ω, e, ħ, τ are, respectively, the angular frequency, electron charge, reduced Planck’s constant and the carrier relaxation time. Moreover, τ is the core loss parameter of graphene, determining the real/imaginary parts of σ and further modulating the reflection coefficients; increasing τ can reduce intraband loss to broaden the waveplate bandwidth. In this study, the ambient temperature T = 300 K and the carrier relaxation time τ = 0.1 ps. The chemical potential $E_{F}$ of graphene can be controlled by applying the bias voltage V_g_ as expressed in Refs. [12,15]:

(2) $$E_{F}≃\hbar v_{f}\sqrt{\frac{\pi \epsilon_{r}\epsilon_{0}V_{g}}{et_{s}}},$$

where $\epsilon_{r},{\;\epsilon }_{0}$ and $v_{f}$ are the relative permittivity of the substrate and vacuum and the Fermi velocity (1.1 × 10^6^ m/s) in graphene, respectively. The substrate dielectric used is silicon dioxide, with a thickness *ts* of 0.1 μm, a dielectric constant $\epsilon_{r}$ of 3.75, and a loss tangent of 0.0004. Using Formula (2), when the Fermi level is 0.9 eV, the required maximum bias voltage is calculated to be 28.7 V, which is within the normal range achievable in a laboratory setting. A thin layer of the transparent conductive oxide (TCO) is applied on the PTFE to serve as the negative electrode of the capacitor structure. Since TCO is extremely thin, its impact in simulations can be negligible [12]. Due to the need for graphene lithography on a corrosion-resistant material, a thin film of silicon dioxide is evaporated onto ITO by electron-beam technology. The modulation of the graphene conductivity can be achieved by applying bias voltage, which can inject both electrons and holes into graphene, allowing for a wide adjustment range of the *E_F_*.

Moreover, the broadband absorption effect is achieved through the plasmonic resonance of VO_2_ and the gold reflective film. By altering the *E_F_* of graphene, the resonant period can be modified, allowing for modulation of both the amplitude and frequency for the absorption spectra. When the temperature is below 300 K, VO_2_ has a monoclinic crystal structure with high resistivity and insulation properties and its conductivity can be entirely restored to its initial state [37]. When the temperature T > 340 K, VO_2_ transforms into a cubic rutile structure with metal properties and its electrical conductivity will increase by 4~5 orders of magnitude in a small change range of the temperature. The phase transition of VO_2_ is reversible. Additionally, its dielectric constant in the terahertz band is also shown by the Drude model [26]:

(3) $$\varepsilon \left(\omega \right)=\varepsilon_{\infty }-\frac{\omega_{p}^{2}\left(\sigma \right)}{\left(\omega^{2}+i\gamma \omega \right)},$$

where the permittivity at infinite frequency $\varepsilon_{\infty }\;=\;12$, collision frequency $\gamma \;=\;5.75\;\times \;10^{13}\;\mathrm{rad}/s,$ and plasma frequency ${\omega_{p}}^{2}\left(\sigma \right)\;=\;\left(\sigma /\sigma_{0}\right){\omega_{p}}^{2}\left(\sigma_{0}\right),\;\mathrm{in}\;\mathrm{which}\;\sigma_{0}\;=\;3\;\times \;10^{5}\;S/m$ and $\omega_{p}\left(\sigma_{0}\right)\;=\;1.4\;\times \;10^{15}\;\mathrm{rad}/s$ [11]. Additionally, γ is the intrinsic loss parameter of metallic VO_2_, governing the dipole resonance damping and total reflection coefficient R; reducing γ weakens resonance loss to expand the absorption bandwidth. In this work, the conductivities of VO_2_ in the broadband absorber and polarization converter modes were $\sigma \;=\;2\;\times \;10^{5}\;S/m\;\left(T_{C}\;=\;340\;K\right)$ and $\sigma \;=\;20\;S/m\;\left(T\;=\;300\;K\right)$, respectively.

Based on the commercial CST software, the reflected wave can be decomposed into the *x*-component and the *y*-component reflection:

(4) $$E_{r}=E_{xr}e_{x}+E_{yr}e_{y}=\left|r_{xx}\right|\exp\left(i\varphi_{xx}\right)E_{xi}e_{x}+\left|r_{yx}\right|\exp\left(i\varphi_{yx}\right)E_{yi}e_{y}.$$

To describe the reflected wave and its polarization state, the Stokes parameters are introduced as Refs. [11,12]:

(5) $$S_{0}\;=\;{\left|r_{\mathrm{xx}}\right|}^{2}\;+\;{\left|r_{\mathrm{yx}}\right|}^{2}$$

(6) $$S_{1}={\left|r_{\mathrm{xx}}\right|}^{2}-{\left|r_{\mathrm{yx}}\right|}^{2}$$

(7) $$S_{2}=2\left|r_{\mathrm{xx}}\right|\left|r_{\mathrm{yx}}\right|\cos\left(\Delta \varphi \right)$$

(8) $$S_{3}=2\left|r_{\mathrm{xx}}\right|\left|r_{\mathrm{yx}}\right|sin\left(\Delta \varphi \right).$$

The Stokes parameters S_0_, S_1_, S_2_ and S_3_ stand, respectively, for the total reflection, the horizontal and vertical linear polarization (LP) state of the reflection, the linear +45-degree or −45-degree polarization state of the reflection, and the CP state. The polarization conversion efficiency (PCR) and the polarization conversion capability characterized by the ellipticity *e* are defined, respectively, as [11]:

(9) $$\mathrm{PCR}=\frac{{\left|r_{yx}\right|}^{2}}{S_{0}}$$

(10) $$e=\frac{S_{3}}{S_{0}},$$

In general, the degree of linear polarization (DoLP) is used to describe the conversion of CP light into LP light, which can be calculated as [11,38]

(11) $$DoLP=\frac{\sqrt{S_{1}^{2}+S_{2}^{2}}}{S_{0}}$$

In studying the polarization conversion characteristics, for computational convenience, the direction of the TE wave is set at +45 degrees along the positive x-axis.

## 3. Results and Discussion

### 3.1. Functionality Switching Between HWP and QWP

**Figure 2:**
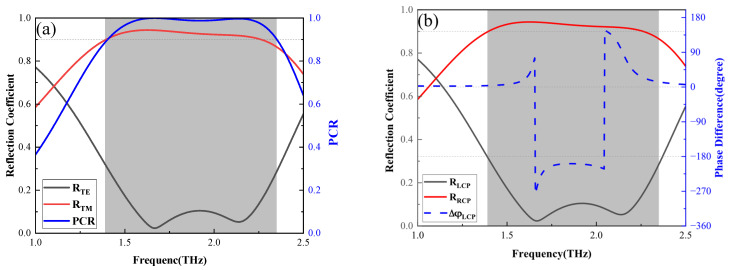
(**a**) Reflection coefficients for TE waves and TM waves and PCR when temperature is under 300 K with an *E_F_* of 0.9 eV for graphene. (**b**) The reflection coefficients for LCP and RCP light, as well as the phase difference between them, when the incident wave is LCP light.

When the temperature is below 300 K, the multifunctional metasurface can switch between HWP and QWP by adjusting the *E_F_* of the graphene. As the *E_F_* of graphene is set to 0.9 eV, the designed multifunctional metasurface achieves cross-polarization conversion, as shown in Figure 2a. One can see that both the PCR in the range of 1.39–2.34 THz and the reflectance of TM waves in the range of 1.39–2.26 THz are greater than 90%. This indicates that the device maintains high polarization conversion efficiency while minimizing losses. In Figure 2b, when the incident wave is set to left-circularly polarized (LCP) light, efficient conversion to right-circularly polarized (RCP) light is achieved in a frequency range of 1.39 to 2.26 THz, with a phase difference of 0 degrees and −180 degrees. This demonstrates that the device performs a HWP function.

**Figure 3:**
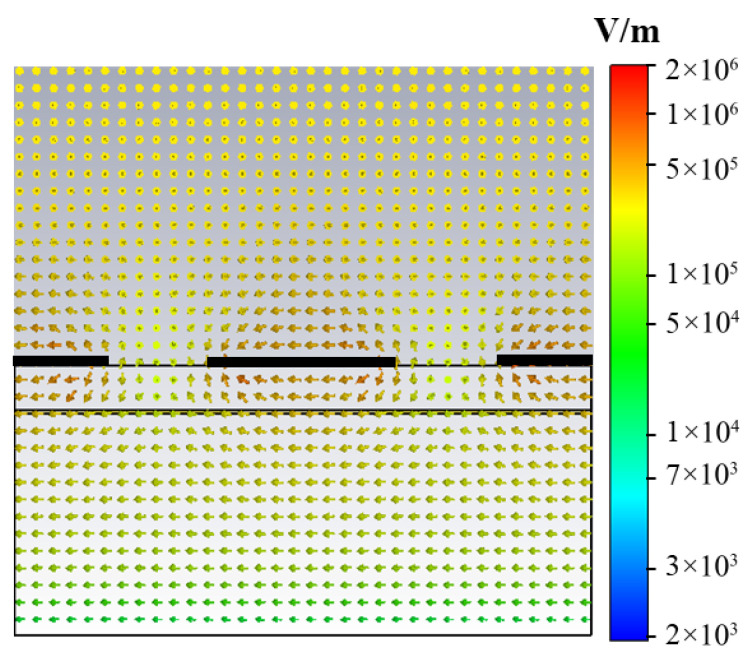
Side view of the current direction for the unit in the y-face, when the TE wave is incident at +45 degrees along the positive x-axis.

The polarization conversion performance of the designed multifunctional metasurface is generated by the anisotropy of the graphene. When the incident light is TE wave at +45 degrees along the positive x-axis and the Fermi level, *E_F_*, of the graphene is 0.9 eV, the direction of the surface electric field in the y-face is as shown in Figure 3. To facilitate observation, the relative position of graphene is specifically depicted with three thick black lines. The three horizontal thin black lines from top to bottom under the thick line represent the graphene structure plane, the vanadium dioxide structure plane, and the gold reflection plane, respectively. The currents are generated between the edges of the adjacent graphene ribbons, causing the conversion of TE waves into TM waves, which can illustrate that the device can function as a half-wave plate.

**Figure 4:**
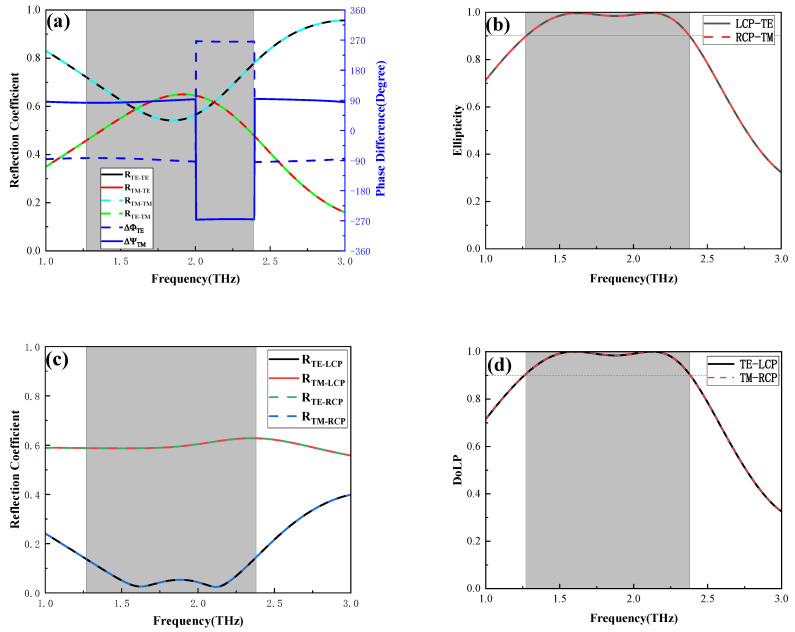
Reflection coefficients, phase difference (**a**) between TE and TM waves and the ellipticity (**b**) of the outgoing light when the incident light is LP and the *E_F_* of the adjacent graphene ribbons is 0.1 eV and 0.6 eV. Reflection coefficients (**c**) for outgoing TE and TM waves and the DoLP (**d**) when the incident wave is CP light.

Moreover, when the *E_F_* of adjacent graphene ribbons is set to 0.9 eV and 0.1 eV, the designed multifunctional metasurface achieves LTC polarization conversion. The conversion of TE waves to TM waves results in the magnitudes of the TE and TM waves both approaching 0.5, with their phase difference close to ±90 degrees or ±270 degrees, facilitating effective conversion to LTC polarization as shown in Figure 4a. At the frequency between 1.27 and 2.38 THz, the ellipticity of the outgoing light exceeds 90% and is close to 1, demonstrating successful LTC polarization conversion in Figure 4b. As shown in Figure 4c,d, when the incident light is LCP, the TM component of the transmitted light approaches 0, resulting in a DoLP greater than 0.9 in the same frequency band. This shows that the designed device can effectively function as a QWP in the frequency range of 1.27–2.38 THz.

**Figure 5:**
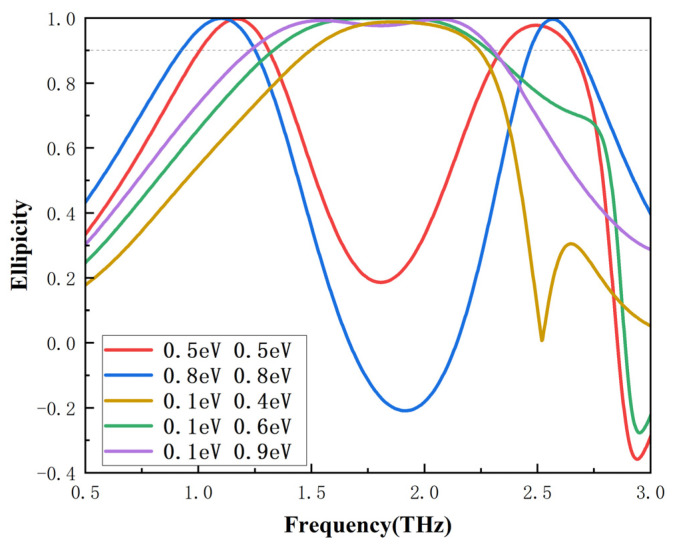
The ellipticity of the outgoing light for graphene at different *E_F_* values of adjacent graphene ribbons.

When the incident light is TE wave, under the conditions of adjacent graphene layers with five sets of different Fermi levels, the ellipticity of the outgoing light will change from a double peak to a single peak, as illustrated in Figure 5. As the Fermi levels of the adjacent graphene strips are set as 0.5 eV and 0.5 eV and 0.8 eV and 0.8 eV, respectively, the ellipticity curves with double peaks are shown with red and blue lines. However, when the Fermi levels of the adjacent graphene strips are chosen as 0.1 eV and 0.4 eV, 0.1 eV and 0.6 eV, and 0.1 eV and 0.9 eV, respectively, the ellipticity curves with a broadband single peak are shown with yellow, green and purple lines; the purple line has a broader operating bandwidth. For the various designed graphene periods, by adjusting the Fermi level of the graphene, one can obtain the nearly complete coverage of 90% LTC polarization conversion in the frequency range from 0.92 to 2.68 THz, which demonstrates the significant practical value of the designed graphene structure.

**Figure 6:**
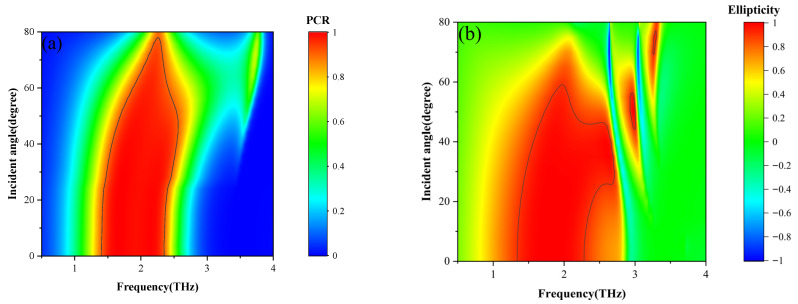
The PCR at different incident angles when the device operates as a HWP (**a**) and the ellipticity at different incident angles when the device operates as a QWP (**b**).

In addition, the impact of incident angle on the waveplate performance of the multifunctional metasurface is also studied. As shown in Figure 6a,b, the PCR and ellipticity at different frequencies are given as the incident angle increases from 0 to 80 degrees. Within the bandwidth indicated by the black lines, both PCR and *e* exceed 0.9, which clearly illustrates that the waveplate performance of the device maintains a high operational bandwidth even under large incident angles of about 50 degrees.

### 3.2. Broadband Switchable Absorber

When the temperature is above 340 K, the VO_2_ layer plays a major role and electric dipole resonance occurs between the VO_2_ layer and the gold layer, leading to absorption effects. In the study of the absorption effects of the multifunctional metasurface, the TE wave direction is parallel to the positive direction of the x-axis. By using a bias voltage to tune the conductivity of graphene, the resonance structure of vanadium dioxide can be changed. This can not only decrease the resonance of the TE wave but also alter the resonance period of the TM wave, causing a red shift in the TM wave’s resonance frequency.

**Figure 7:**
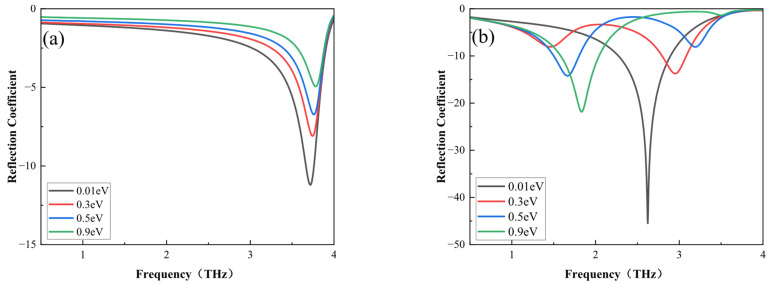
Reflection coefficients for TE waves (**a**) and TM waves (**b**) versus the *E_F_* of graphene at T = 340 K.

The reflection coefficients were logarithmically transformed with a base of 10 and then multiplied by 20. In Figure 7a, the amplitude of the TE-polarized waves increases from −11.32 dB to −4.90 dB with the rise in the *E_F_.* In Figure 7b, the amplitude of the TM-polarized waves increases, and the resonance frequency gradually red-shifts from 2.61 THz to 1.81 THz with an increase in the E_F_. The modulation depth is defined as MD = (T_on_−T_off_)/T_on_ [22]. If we assume the on and off states to be E_F_ = 0.9 and 0.01 eV, respectively, the proposed modulator achieves a high modulation depth of 96.9% at 2.61 THz, indicating substantial potential for the device as an amplitude modulator. In Figure 7, one can ascertain that the bandwidth of the absorptivity exceeding 90% for TM waves gradually transitions from 2.28–3.12 THz to 1.41–2.04 THz. Furthermore, the absorptivity can exceed 90% at the frequency between 1.41 and 3.12 THz by adjusting various graphene bias voltages, suggesting wideband functionality. However, the absorptivity for TE waves can exceed 90% in the 3.65–3.78 THz range.

**Figure 8:**
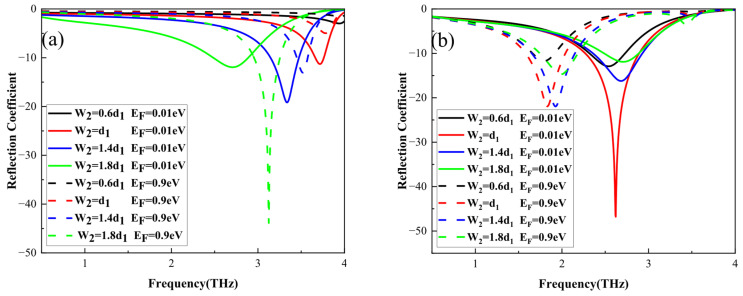
The reflection coefficients of TE waves (**a**) and TM waves (**b**) under the different w_2_ and graphene *E_F_* values when w_1_ = 1.8d_1_ = 28.8 μm.

By optimization when w_1_ = 1.8d_1_ = 28.8 μm, the effects of the width w_2_ and *E_F_* of graphene on the reflection coefficients of the TE waves and TM waves are also analyzed, as shown in Figure 8. One can see that the reflection coefficients of the TM waves at *E_F_* = 0.01 eV and 0.9 eV and the reflection coefficients of the TE waves at *E_F_* = 0.01 eV decrease initially, then increase with an increase in w_2_, while the reflection coefficients of the TE waves at *E_F_* = 0.9 eV always decrease. In Figure 8a, the resonant frequency of the absorption peak for the TE waves indicates a gradual red shift as w_2_ increases, while the absorption frequency displays a gradual blue shift as *E_F_* increases. However, in Figure 8b, the change in the resonant frequency for the TM waves with the increase in w_2_ and *E_F_* is the opposite of the TE waves and is not much. Thus, one can also see that the optimal modulation depth and maximum absorptivity are 96.9% and 99.6% at w_2_ = d_1_, because of the electric dipole resonance between the VO_2_ layer and the gold reflective film.

### 3.3. The z-Component Distribution of the Surface Electric Field

**Figure 9:**
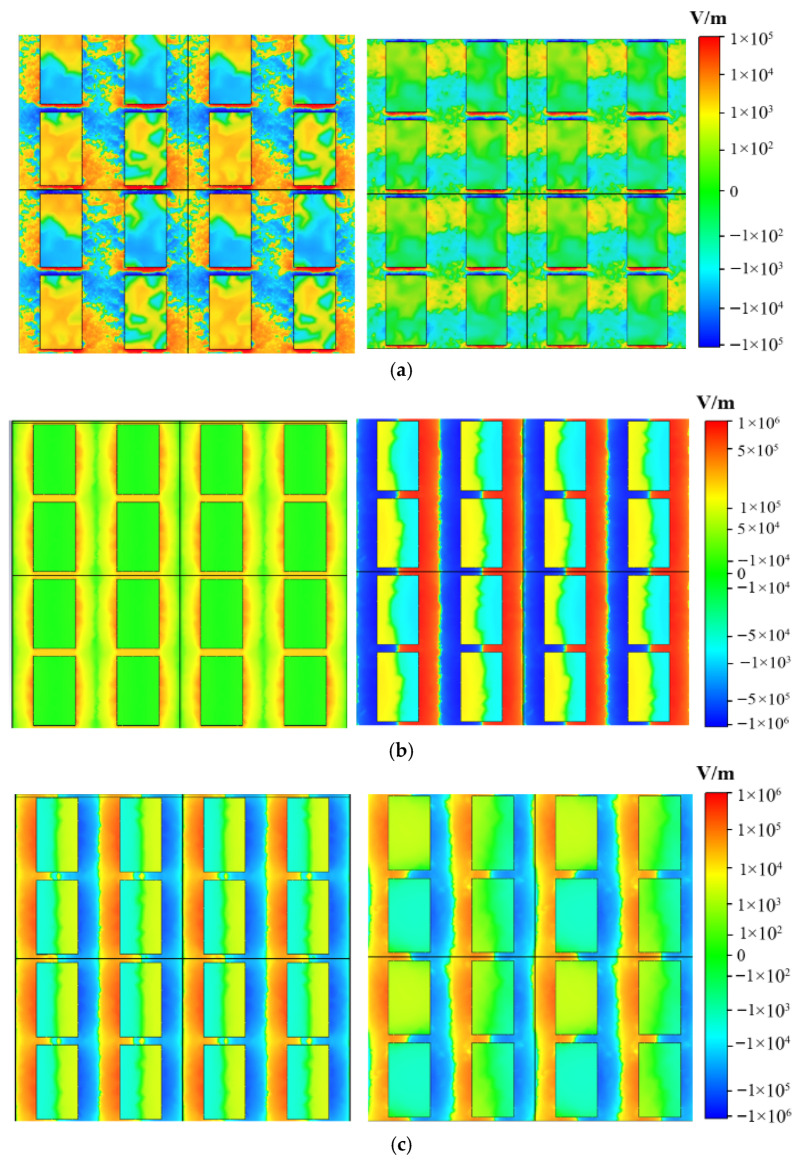
The z-component of the surface electric field of VO_2_ at different absorption peaks when the *E_F_* of graphene varies from 0.01 eV (**left**) to 0.9 eV (**right**) at 3.72 THz (**a**), 2.61 THz (**b**), and 1.81 THz (**c**).

The z-component of the surface electric field of VO_2_ at 3.72 THz for TE-polarized wave incidence is depicted in Figure 9a. On the left and right sides, the *E_F_* values of the graphene are 0.01 eV and 0.9 eV, respectively. It is observed that the electric field primarily concentrates on the upper and lower ends of the unit rectangle, with the opposite polarities at the top and bottom. This results from the dipole resonance of the rectangular aperture VO_2_ and the gold reflective film, leading to an absorption peak. Similarly, Figure 9b,c illustrates the distribution of the z-component of the surface electric field for the TM-polarized waves at 2.61 THz and 1.81 THz, respectively. It is evident that the device absorptivity for multiple-frequency bands can be adjusted under the control of the bias voltage.

**Figure 10:**
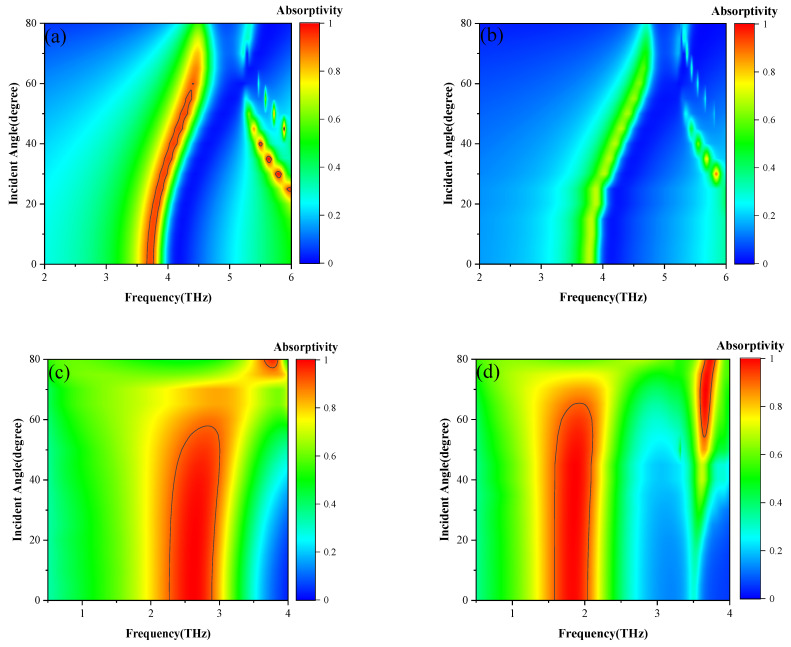
The absorptivity of TE waves when *E_F_* is 0.01 eV (**a**) and 0.9 eV (**b**) and the absorptivity of TM waves when *E_F_* is 0.01 eV (**c**), and 0.9 eV (**d**).

Simultaneously, the impact of the incident angle on the dual-parameter absorption of the multifunctional metasurface is also investigated. The absorptivity can be directly calculated as $A=1-R=1-{\left|r_{xx}\right|}^{2}-{\left|r_{yx}\right|}^{2}$ [11]. Figure 10 illustrates the peak absorption spectra of TE waves and TM waves for the metasurface device when the *E_F_* of the graphene is 0.01 eV and 0.9 eV, respectively. It can be observed in Figure 10a,b that the absorptivity of the TE waves remains high at incident angles less than 50°, while it decreases sharply at angles larger than 50°. The absorption at the higher angles is reduced due to the broken phase-matching of VO_2_–gold dipole resonance and anisotropic propagation of graphene SPPs, and the higher E_F_ of 0.9 eV increases graphene surface reflection, leading to the lower overall absorptivity. While in Figure 10c,d, one can see that after the modulation of the graphene, the absorption band of the TM waves undergoes a red shift, satisfying the conditions for large-angle incidence.

### 3.4. Performance Comparison

Finally, our designed metasurface is compared with the recently reported works in Table 1. Most of them exhibit high performance in the individual functionalities, and some have specific manipulation characteristics, but not within this terahertz frequency band. The proposed metasurface features a relatively simple structural design, and achieves three distinct functionalities of HWP, QWP, and absorber. Moreover, all these functionalities can be rapidly modulated through bias voltage, indicating promising prospects for broader applications.

## 4. Conclusions

In this study, we propose a multifunctional terahertz metasurface device based on graphene, capable of transitioning between waveplates and absorbers by changing the bias voltage and temperature. When the temperature is below 300 K and *E_F_* = 0.9 eV, the device functions as a HWP, achieving a PCR greater than 90% in the range of 1.39–2.34 THz. With adjacent graphene bands at *E_F_* values of 0.1 eV and 0.9 eV, the device operates as a QWP, exhibiting ellipticity greater than 90% in the range of 1.27–2.38 THz. Moreover, at the different *E_F_* values, the device almost universally achieves QWP functionality in the range of 0.92–2.68 THz. Under conditions of temperature exceeding 340 K, the device switches to an absorber, allowing the dual modulations of TE wave amplitude and TM wave resonance frequency. As the *E_F_* transitions from 0.01 eV to 0.9 eV, the TE wave at 3.72 THz undergoes absorption modulation from −11.32 dB to −4.9 dB, while the TM wave resonance frequency shifts from 2.61 THz to 1.81 THz, achieving a modulation depth of 96.9% at 2.61 THz. Therefore, this versatile metasurface with a broad operating frequency bandwidth and high efficiency can be employed in constructing terahertz communication systems, polarizing devices, and modulators.

## Figures and Tables

**Table 1:** The comparison between our work and the other reports.

Ref.	Active Materials	Dynamic Switching of HWP and QWP in the Same Frequency Band	Waveplate Dynamic Tuning	Parameter Control	The Modulation Method of the Absorber
[39]	VO_2_	Only HWP (0.82–1.60 THz)	NA	Only one parameter 0.68–1.60 THz	VO_2_
[40]	VO_2_	Only HWP (0.42–1.04 THz)	NA	Only one parameter 0.52–1.20 THz	VO_2_
[41]	VO_2_	Only QWP (1.47–2.27 THz)	NA	Only one parameter 0.74–1.62 THz	VO_2_
[32]	VO_2_ and photo- sensitive silicon	Only HWP (0.51–1.45 THz)	NA	Only one parameter 0.78–1.81 THz	VO_2_ and photosensitive silicon
[11]	VO_2_ and graphene	HWP(0.68–2.64 THz) QWP(0.89–2.51 THz)	NA	Only one parameter 0.89–2.36 THz	VO_2_ and graphene
[23]	Graphene	NA	NA	Two parameters TE: 4.65 GHz TM: 3.35–4.60 GHz	graphene
This work	VO_2_ and graphene	HWP(1.39–2.34 THz) QWP(0.92–2.68 THz)	Yes	Two parameters TE: 3.65–3.78 THz TM: 1.41–3.12 THz	VO_2_ and graphene

## Data Availability

The data presented in this study are available on request from the corresponding author due to privacy restrictions of the research group. If there are any questions about the data shown in the manuscript, please contact the corresponding authors.
